# Advanced biomaterial strategies for cancerous wound management from palliative to multifunctional platforms

**DOI:** 10.1016/j.isci.2026.115811

**Published:** 2026-04-20

**Authors:** Shuang Ma, Chi Wang, Meiqi Li, Shitong Zhang, Cui Wang, Fang Ma, Yuan Zheng, Dan Yang

**Affiliations:** 1 The Fourth Affiliated Hospital of China Medical University, Shenyang, Liaoning 110032, China; 2 Liaoning Normal University, Shenyang, Liaoning 116029, China

**Keywords:** Oncology, Supportive care, Biomaterials

## Abstract

Cancerous wounds cause severe pain, odor, exudate, and bleeding, severely impairing patients’ quality of life. Traditional management focuses on palliative symptom control. However, advances in biomaterials, drug delivery, and immunomodulation are transforming wound dressings from passive coverings into multifunctional therapeutic platforms. This review systematically outlines contemporary strategies for cancer wound management, covering evidence-based conventional dressings and emerging smart technologies, including injectable hydrogels, nanocomposites, and bioactive materials. These innovations not only control core symptoms but also demonstrate local immune modulation and antitumor activity. The paper further analyzes clinical translation challenges and outlines future directions, aiming to advance cancer wound management from palliative care toward integrated therapeutic models.

## Graphical abstract

**Figure:**
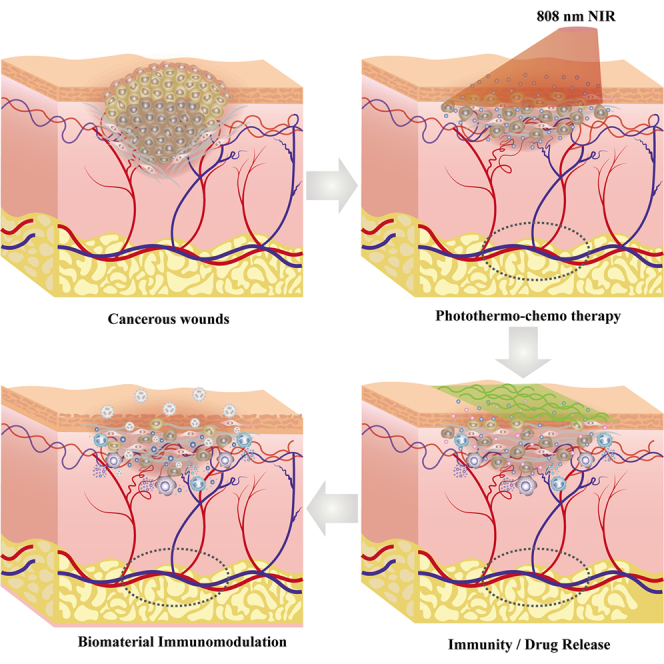

## Introduction

Cancerous wounds typically refer to non-healing lesions formed when primary cutaneous malignancies or metastatic cancers invade and destroy skin tissue. They serve not only as a visible sign of malignant tumor progression but also as a primary cause of severely diminished quality of life for patients.^1^^,^^2^ The formation of cancerous wounds begins when malignant tumor cells acquire invasiveness through epithelial-mesenchymal transition (EMT), breaching the basement membrane and invading the dermis and subcutaneous tissue. During this process, excessive expression of vascular endothelial growth factor (VEGF) leads to the formation of numerous structurally abnormal and dysfunctional neovascularization within the tumor.^3^ Combined with the tumor’s rapid growth exceeding the limits of blood supply, this results in tissue hypoxia and widespread necrosis. These abnormal pathobiological mechanisms interact to form a “vicious cycle” that impedes healing and promotes destruction. In contrast to the orderly repair process of normal wound healing, cancerous wounds remain in a state of continuous and uncontrolled destruction.^4^ Cancerous wounds, typically stage III/IV, arise from primary tumor invasion or cutaneous metastasis—most often in skin and breast cancers but any type—and are accelerated by comorbidities that impair tissue integrity and repair.

The complex pathobiological processes underlying cancerous wounds determine their fundamental differences in clinical presentation, treatment response, and patient prognosis, representing a challenging intersection between oncology and wound care.^5^ Uncontrollable malodor, excessive exudate, recurrent bleeding, and severe pain constitute the classic “four major symptoms” of cancer wounds .^6^ Here, the traditional goal of wound healing shifts to “palliative control”—managing symptoms, preventing complications, and preserving patient dignity and quality of life.^7^ However, symptom control itself presents inherent contradictions and limitations in therapeutic strategies. For instance, how does one balance hemostasis with avoiding tissue necrosis?^8^ How does one manage the interactions between wound healing and antitumor therapies such as radiotherapy and chemotherapy ?^9^ Simultaneously, the heavy burden of frequent dressing changes, complex dressing selection, and odor management severely erodes patient dignity and quality of life, creating immense caregiving burdens and psychological distress.^10^^,^^11^^,^^12^

Cancer wounds represent a complex clinical challenge driven by unique pathobiology, exhibiting devastating clinical manifestations and profoundly detrimental effects on patients’ quality of life. A thorough understanding of the underlying “vicious cycle” biological mechanisms is fundamental to exploring effective management strategies.^13^^,^^14^ This paper aims to systematically review the latest advances in the pathobiology of cancer wounds. Building upon this foundation, it delves into the current clinical management challenges and emerging palliative care and supportive nursing strategies, with the goal of providing theoretical underpinnings and practical guidance for improving patient survival quality.^15^

Achieving this vision requires a truly multidisciplinary effort. Materials scientists and biomedical engineers are developing novel polymers, nanostructures, and hydrogels for wound scaffolds, while cancer biologists and pharmacologists identify potent antitumor agents for local delivery. Clinical oncologists and palliative specialists contribute insights on tumor biology, wound symptomatology, and patient care needs. As one systematic review emphasizes, only an “interdisciplinary and individualized treatment strategy” can adequately address the complex symptoms of cancerous wounds. In this light, the innovations at the intersection of nanotechnology, biomaterials science, drug delivery, and immunotherapy are critical. In the following sections, we will survey the state-of-the-art classes of advanced dressings—including nanofiber meshes, drug-loaded hydrogels, and immunomodulatory matrices—that exemplify this paradigm shift. Each of these emerging platforms illustrates how cancer wound care is evolving from mere palliation toward comprehensive, tumor-directed therapy, integrating multiple disciplines to improve outcomes in this challenging setting.

## Pathobiological mechanisms of cancerous wounds

**Figure 1:**
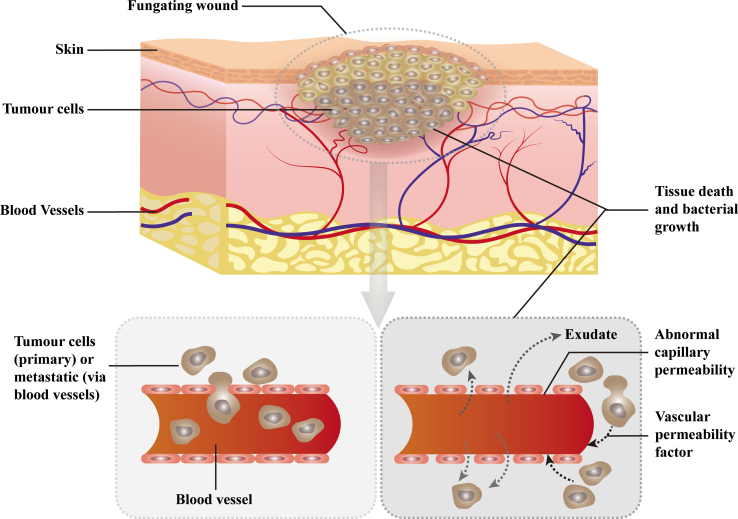
Diagram represents a malignant fungating wound

Cancerous wounds arise in advanced cancer when tumors invade the skin and blood vessels, leading to tissue necrosis, inflammation, and abnormal neovascularization.^16^^,^^17^^,^^18^ This results in non-healing ulcers characterized by bleeding, malodor, and exudate, as the normal, orderly process of tissue repair is hijacked and disrupted by the invading tumor cells and the pathological tumor microenvironment. The key pathophysiological mechanisms involve cancer cell infiltration that disrupts the blood supply, creating hypoxic and necrotic tissue.^19^^,^^20^ This environment is prone to infection, particularly with odor-producing anaerobes. The condition causes severe physical symptoms and considerable psychosocial distress. As curative options are often limited, palliative care focuses on managing symptoms such as pain, odor, and bleeding. The essence of cancerous wounds lies in the “hijacking” and “distortion” of normal wound healing processes by tumor cells. Their complexity stems from the co-drive of abnormal pathobiological mechanisms (Figure 1).

The designation of cancerous wounds as a “palliative” condition stems from the significant limitations and risks associated with applying standard curative-intent anticancer therapies in this specific context. While treatments such as radical surgery, high-dose radiotherapy, and systemic chemotherapy are the mainstay for managing localized or advanced cancers, their application becomes extraordinarily complex once a malignant wound has formed. The presence of a friable, infected, and non-healing ulcer fundamentally alters the risk-benefit calculus, often rendering these standard approaches ineffective, inadvisable, or even harmful. This therapeutic deadlock necessitates a shift in the primary goal of intervention from cure to the optimization of quality of life through meticulous symptom control.

**Figure 2:**
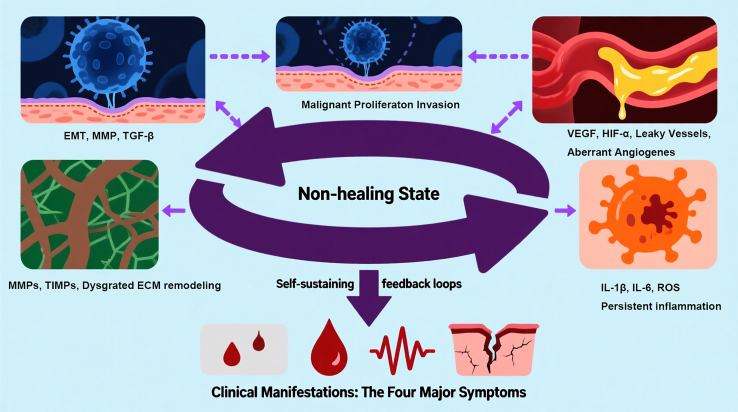
Core pathobiological mechanisms driving cancerous wounds Four interconnected processes—malignant invasion, aberrant angiogenesis, persistent inflammation, and dysregulated ECM remodeling—form self-sustaining feedback loops (purple arrows) that perpetuate the non-healing “vicious cycle” (center). These mechanisms collectively give rise to the characteristic clinical symptoms: exudate, bleeding, pain, odor, and non-healing ulceration (bottom).

A comprehensive understanding of the pathobiological mechanisms underlying cancerous wounds is essential for developing effective management strategies. Unlike acute wounds that follow an orderly healing trajectory, cancerous wounds are perpetuated by a complex interplay of tumor-driven processes that subvert normal tissue repair. These include uncontrolled malignant proliferation, aberrant angiogenesis, persistent inflammation, and dysregulated extracellular matrix (ECM) remodeling. Each of these interconnected mechanisms not only contributes to the destructive phenotype but also generates self-sustaining feedback loops that render the wound chronically non-healing. Elucidating these pathways provides the rational foundation for both current symptom-oriented interventions and the development of next-generation therapeutic dressings discussed in subsequent sections. The following subsections detail the four core pathological processes that collectively drive the vicious cycle of cancerous wounds (Figure 2).

### Malignant proliferation and invasion of cancer cells

Cancer cells divide uncontrollably, proliferating at a rate far exceeding apoptosis, directly disrupting the normal tissue architecture of the skin.^21^ Furthermore, they release various hydrolases such as matrix metalloproteinases (MMPs),^22^^,^^23^ which act like “scissors” to degrade the ECM, clearing obstacles for their own spread. Simultaneously, through complex cellular signaling pathways, cancer cells disrupt the functions of surrounding normal repair cells such as fibroblasts.^15^ This prevents them from synthesizing new collagen and tissue frameworks, thereby inhibiting the initiation of effective wound repair processes.^2^^,^^14^ Research reveals that the squamous cell carcinoma antigen SerpinB3 plays a pivotal role in this process. Acting as a stress response factor to epithelial injury, it induces keratinocytes to undergo EMT-like phenotypic changes.^16^^,^^24^ This may be one of the primary reasons cancer cells possess formidable migration and invasion capabilities.^25^ It is precisely this hijacked, abnormal “pseudo-healing” mechanism that prevents normal, orderly epithelialization at the edges of cancerous wounds. Instead, it promotes cancer cell dissemination, keeping the wound in a state of persistent ulceration and continuous expansion.^26^^,^^27^^,^^28^

### Abnormal angiogenesis

Tumor cells secrete high levels of VEGF, excessively stimulating and inducing the proliferation of numerous structurally abnormal, dysfunctional new blood vessels.^29^ These vessels are not normal mature vessels; they are tortuous and dilated, with incomplete wall structures and discontinuous basement membranes, resembling substandard pipes. The high permeability of these vessel walls allows substantial plasma components to leak out, resulting in persistent serous exudation at the wound site and severe edema in surrounding tissues.^30^^,^^31^ Simultaneously, this leakage impairs blood flow and oxygen supply, exacerbating the vicious cycle of tissue hypoxia. Furthermore, the fragile vessel walls cannot withstand even minor physical stress and rupture easily, leading to frequent spontaneous or contact bleeding at the wound site. This is the fundamental reason for the massive exudation and high bleeding risk in cancerous wounds, posing significant challenges for treatment and care.^32^^,^^33^

### Persistent chronic non-healing inflammation

Unlike the transient and controllable inflammatory response during normal wound healing, the microenvironment of cancerous wounds is chronically saturated with abundant immune cells (such as macrophages and neutrophils) and inflammatory mediators (such as TNF-α, IL-1β, and IL-6), creating a highly active and destructive inflammatory environment.^34^ This chronic inflammation acts like a double-edged sword.^25^^,^^33^ On one hand, it directly exacerbates local tissue damage and necrosis, triggering intense pain and producing a distinctive foul odor.^35^^,^^36^ On the other hand, these inflammatory factors transform into signals stimulating tumor growth, acting like “fertilizer” to further nourish and activate tumor cells, promoting their proliferation and invasion. This creates a self-reinforcing “inflammation-tumor” vicious cycle, causing the wound to progressively deteriorate.^37^ Research indicates that metabolic imbalance of hyaluronic acid (HA)—a crucial component of the ECM—plays a pivotal role in this process. High-molecular-weight hyaluronic acid (HMW-HA), which inherently possesses anti-inflammatory properties, undergoes extensive degradation in cancerous wounds. The resulting low-molecular-weight hyaluronic acid (LMW-HA) exhibits potent pro-inflammatory characteristics, significantly exacerbating this vicious cycle.^38^^,^^39^

### Imbalanced extracellular matrix remodeling

**Figure 3:**
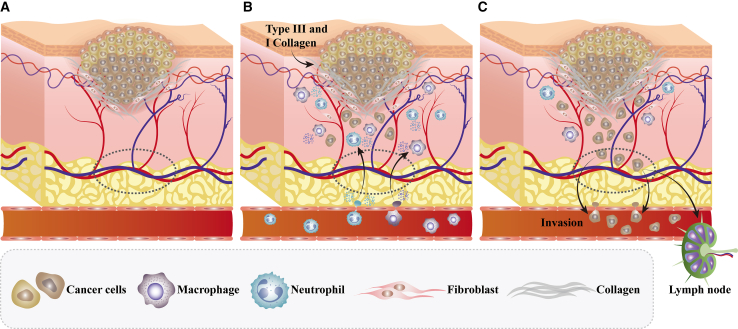
Schematic representation of an epithelial tumor wound (A) When neoplasia is first initiated, fibroblasts are recruited to the tumor site and activated. (B) As the tumor grows, inflammatory cells are recruited to the tumor and release cytokines. VEGF and other signaling molecules induce neovascularization. (C) The abnormal extracellular matrix is pro-tumorigenic, pro-angiogenic, and increases the invasiveness of the tumor.

The normal ECM serves as the “scaffolding” for tissue repair. However, in cancerous wounds, the expression balance between MMPs such as MMP-2 and MMP-9 and tissue inhibitors of metalloproteinases (TIMPs) is disrupted (Figure 3). Excessively secreted MMPs, acting like “runaway molecular scissors,” cause excessive and disorderly degradation of normal ECM components—such as structurally intact type I and III collagen, elastic fibers that provide tissue elasticity, and fibronectin that promotes cell adhesion.^40^^,^^41^ This not only directly disrupts tissue structural integrity—rendering wound margins fragile and incapable of closure, thereby creating pathways for malignant tumor cell invasion—but also triggers massive release of bioactive factors such as endostatin, interfering with normal angiogenesis. More critically, the tumor microenvironment deposits a disordered, rigid, and dysfunctional ECM.^42^^,^^43^^,^^44^ This remodeled abnormal ECM not only fails to transmit proper repair signals but also actively drives tumor cell proliferation and invasion by conveying abnormal mechanical and biochemical signals to cells via pathways such as integrins. Simultaneously, it suppresses normal fibroblast function, ultimately locking the wound site in a vicious cycle of persistent destruction and inability to initiate repair processes. This constitutes one of the fundamental reasons for the poor healing of cancerous wounds.^45^^,^^46^^,^^47^

## Clinical strategies for managing cancer wound symptoms with conventional dressings

In the management of cancerous wounds, the core objective of dressing application has shifted from pursuing healing to palliative care focused on symptom control. Moving beyond traditional dressing concepts, the current emphasis lies in scientifically and precisely selecting and applying dressings based on the clinical symptoms of cancerous wounds, their unique pathophysiological mechanisms, and palliative care goals.^48^^,^^49^^,^^50^ See Table 1.

**Table 1:** Clinical dressing selection strategy for symptom management in cancer wounds: Mechanisms, rationale, and evidence

Symptom	Recommended dressing types	Mechanism of action	Clinical rationale (basis for recommendation)	Key references
Malodor	metronidazole gel (0.75%–0.8%)	nitro group reduction creates cytotoxic metabolites that disrupt anaerobic bacterial DNA.	anaerobes are primary producers of volatile malodorous compounds (e.g., putrescine and cadaverine). Topical metronidazole targets the root cause.	Scheer et al.^51^
	silver-containing dressings	silver ions (Ag+) disrupt bacterial cell membranes, bind to DNA, and interfere with metabolic enzymes.	provides broad-spectrum coverage including both aerobes and anaerobes; synergistic with metronidazole.	Gadre et al.^48^, Scheer et al.^51^
	activated charcoal dressings	physical adsorption of volatile odor molecules through high-surface-area porous carbon.	functions as a secondary dressing to capture odors at the surface; efficacy diminishes in high exudate; requires combination with antimicrobials.	Probst et al.,^52^ Tsai et al.,^53^ Saha et al.^54^
	medical-grade honey (e.g., Manuka)	hyperosmotic environment, low pH, sustained H_2_O_2_ release, and methylglyoxal (MGO) activity.	multi-mechanism antimicrobial with anti-biofilm properties; provides comfort and autolytic debridement.	Pavel et al.,^3^ Feng et al.^55^
Exudate (High Volume >10 mL/10 cm^2^/day)	alginate dressings (calcium alginate)	ion exchange (Ca^2+^ for Na^+^) forms a hydrophilic gel; absorbs 20–30× its weight.	high absorbency for moderate-to-heavy exudate; calcium release also provides hemostatic benefit (see later in discussion).	Khaliq et al.,^56^ Rajput et al.^57^
	hydrofiber dressings (sodium carboxymethylcellulose)	forms cohesive gel on contact with exudate; locks fluid into dressing structure.	superior fluid retention minimizes maceration of periwound skin; ideal for irregular wounds.	Khaliq et al.^56^
	foam dressings (polyurethane/silicone)	hydrophilic matrix absorbs fluid; hydrophobic outer layer prevents strike-through.	maintains moist wound bed while keeping surrounding skin dry; provides cushioning and reduces pain.	Yuan et al.^58^
	hydrocolloid dressings	gel formation upon exudate absorption; occlusive backing.	suitable for viscous exudate with slough; promotes autolytic debridement.	Koehler et al.,^59^ Liang eta al,^60^ Purushotham et al.^61^
	wound drainage bags/NPWT	passive collection (bags) or active suction (NPWT).	for extremely high output; NPWT requires careful assessment due to bleeding risk.	Probst et al.,^62^ Dvorak et al.^63^
Bleeding	soft silicone contact layer dressings	non-adherent silicone interface minimizes trauma upon removal.	prevents bleeding by avoiding the mechanical disruption of fragile tumor neovasculature during dressing changes.	Firmino et al.^2^
	alginate dressings	calcium ions activate the coagulation cascade (prothrombotic); the physical matrix supports clot formation.	provides both chemical (Ca^2+^) and physical hemostasis; apply with gentle pressure for 10–15 min for active minor bleeding.	Amante et al.,^8^ Khaliq et al.^56^
	hemostatic dressings (e.g., oxidized regenerated cellulose)	physical matrix and activation of platelet aggregation.	for more significant bleeding, a tiered approach from prevention to active management.	Pereira et al.^15^
Pain	non-adherent silicone dressings	minimizes adhesion to wound bed and nerve endings.	reduces procedural pain during dressing changes (the most distressing aspect for patients).	Firmino et al.,^2^ Tamai et al.^7^
	hydrogels (amorphous or sheet)	high water content provides a cooling sensation; maintains a moist environment.	soothing effect on painful, inflamed wounds; facilitates autolytic debridement.	Prabhakar et al.^64^
	lidocaine-infused dressings	local anesthetics block voltage-gated sodium channels in nerve endings.	provides sustained topical analgesia post-dressing change; part of multimodal pain management.	Tamai et al.^7^
Infection	silver ion dressings (multiple formulations)	multifactorial antimicrobial action (see above).	broad-spectrum coverage; available in multiple platforms (alginate, foam, charcoal) for combination with other symptom management.	Gadre et al.,^48^ Scheer et al.^51^
	medical-grade honey	multifactorial antimicrobial (see above).	effective against biofilms; non-cytotoxic to wound bed; promotes autolytic debridement.	Pavel et al.,^3^ Feng et al.^55^
	iodine-based dressings (cadexomer iodine, povidone-iodine)	iodine penetrates microbial cell walls and disrupts protein synthesis.	sustained release formulations provide prolonged antimicrobial activity; effective against MRSA and VRE.	N/A

### Integrated strategy for antimicrobial and odor control

Foul odor is one of the most devastating symptoms of cancerous wounds, primarily arising from tumor tissue necrosis complicated by bacterial infection.^25^ This unbearable stench not only inflicts profound shame and social isolation upon patients, severely compromising their mental health and quality of life, but also presents an urgent challenge in nursing care. Modern odor management emphasizes a multi-tiered, integrated strategy.^24^^,^^33^

Metronidazole, as the gold standard drug for treating anaerobic infections, acts by reducing its nitro group to form cytotoxic metabolites that disrupt the DNA structure of anaerobic bacteria. Studies indicate that daily topical application of 0.75%–0.8% metronidazole gel reduces malodor intensity by 60%–80%. Notably, combining metronidazole with silver ion dressings produces a synergistic effect. The latter exhibits broad-spectrum activity against both Gram-positive and Gram-negative bacteria by disrupting bacterial cell membranes and interfering with metabolic enzyme systems.^51^

Activated carbon dressings capture volatile odor molecules through physical adsorption mechanisms.^52^ However, their adsorption capacity reaches saturation within 24–48 h, and hyper-exudate environments further diminish their efficacy. Therefore, activated carbon dressings are recommended as secondary dressings for use in combination with primary antimicrobial dressings.^53^^,^^54^

Honey dressings exert antimicrobial effects through a hypertonic, low-pH environment and sustained release of hydrogen peroxide. Medical-grade Manuka honey also contains unique methylglyoxal (MGO), which enhances penetration through biofilms.^55^ Randomized controlled trials demonstrate that honey dressings are non-inferior to metronidazole gel in controlling malodorous odors and reducing dressing change frequency, while offering greater patient comfort.^3^

In summary, modern malodor management emphasizes a multi-tiered, multi-modal integrated strategy. Rather than relying on a single approach, it combines mechanical debridement (removal of necrotic tissue), highly absorbent dressings (such as activated charcoal dressings that trap odor molecules), topical antimicrobial agents, and specialized deodorizing products. This comprehensive approach aims to control infection at its source and neutralize odors, thereby restoring patients’ dignity and enhancing their quality of life.^37^^,^^40^

### Precision solutions for exudate management

High exudate volume in cancer wounds (typically exceeding 10 mL/10 cm^2^/day) primarily stems from tumor-associated inflammation. This inflammatory response releases multiple vasoactive substances (such as VEGF and histamine), causing a sharp increase in local vascular permeability and extensive extravasation of plasma components.^65^ This not only leads to tissue edema and skin maceration but also heightens the risk of infection and pain. Effective exudate management requires selecting appropriate dressings based on exudate characteristics.^13^

Alginate and hydrophilic fiber dressings, made from calcium alginate or sodium carboxymethylcellulose, form a hydrophilic gel upon contact with exudate. They can absorb 20–30 times their own weight in fluid while maintaining a moist environment, making them suitable for wounds with moderate to heavy exudate.^56^^,^^57^ The novel silver-ion alginate dressing combines high absorbency with sustained antimicrobial activity. Studies demonstrate that it significantly reduces infection rates and exudate volume in cancer-related wounds.^8^^,^^48^

Foam dressings are made from polyurethane or silicone polymers, offering exceptional absorbency and moisture retention. They effectively maintain a moist wound bed while keeping the surrounding skin dry.^58^ The soft outer layer provides cushioning to reduce patient pain and discomfort, and minimizes dressing changes, making them an ideal choice for managing high exudate levels in cancer wounds.

Hydrocolloid dressings form a gel on the wound surface by absorbing exudate, gently softening, and dissolving necrotic tissue.^59^ This creates a moist, occlusive healing environment that effectively manages exudate while facilitating autolytic debridement. For cancerous wounds with viscous exudate accompanied by sloughing necrotic tissue, hydrocolloid dressings are an ideal choice.^60^^,^^61^

Wound drainage bags or negative pressure wound therapy (NPWT) are more suitable for cancerous wounds with extremely high exudate levels. As a passive collection device, wound drainage bags enable continuous drainage, making them ideal for patients with unstable physical conditions or those receiving home care.^62^ NPWT, however, represents an active solution that efficiently removes exudate, reduces tissue edema, and stimulates granulation tissue growth. Its application requires rigorous assessment, focusing on tumor type, blood supply to the wound site, and potential bleeding risks to prevent serious complications such as vascular rupture or tumor dissemination.^63^

Effective and precise exudate management is central to the care of cancer wounds. The key lies in scientifically selecting dressings based on the characteristics of the exudate, such as its volume and viscosity. For copious thin exudate, highly absorbent alginate dressings or foam dressings may be used. For viscous exudate with tissue sloughing, hydrocolloid dressings may be required to soften and facilitate autolytic debridement.^66^ The fundamental goal is to maintain a moist and balanced wound bed while protecting surrounding skin, thereby enhancing patient comfort and improving quality of life.

### Preventive management of bleeding and pain

Bleeding and pain are the two primary challenges associated with cancerous wounds. Bleeding primarily stems from abnormal tumor neovascularization. These vessels exhibit structural abnormalities and thin, fragile walls, rupturing easily upon minor friction or spontaneous necrosis (Table 2). Pain mainly results from nerve compression caused by direct tumor infiltration and mechanical irritation from dressing adhesion during wound care. Effective prevention is the key strategy.^2^

**Table 2:** Technical characteristics of novel cancer wound dressing platforms

Technology platform	Core materials	Mechanism of action	Key references	Research stage	Advantages
Injectable Hydrogel	hyaluronic acid, chitosan, polyethylene glycol	*in situ* formation, sustained drug release, microenvironment-responsive delivery	Amante et al.,^8^ Rajput et al.,^57^ Palani et al.^67^	preclinical to early clinical	adaptable to complex wound shapes; prolonged drug retention, and reduced dosing frequency
Nanocomposite Dressing	metal nanoparticles, liposomes, polymer nanoparticles (e.g., PLGA), mesoporous silica	enhanced drug delivery, synergistic therapy (e.g., photothermal-chemo), multifunctional integration	Moradian et al.,^49^ Naseri et al.,^68^ Zreiqat et al.^69^	preclinical	high drug loading capacity; controlled release; integration of multiple therapeutic modalities
Bioactive Materials	bioactive glass, silicate ceramics (e.g., Sr-Zn-Si), cerium-doped nanoparticles	ion release (Si, Ca, Sr, Cu, Ce), immune modulation (M1→M2 macrophage polarization), ROS scavenging, tissue regeneration	Mehrabi et al.,^70^ Kim et al.,^71^ Lay-Flurrie et al.^72^	some products commercially available	inherent bioactivity; no drug resistance; accelerated healing; dual antibacterial/antitumor effects
Smart Response Systems	pH-sensitive polymers, enzyme-sensitive peptides, temperature-responsive hydrogels	microenvironment-triggered drug release (pH, MMPs, glucose), on-demand therapy	Palani et al.,^67^ Furka et al.^19^	preclinical	precise drug delivery; maximized efficacy; minimized systemic toxicity
3D-Printed Scaffolds	bioinks, cell-laden hydrogels, polymer blends (e.g., PCL/gelatin)	precise structural control, cell delivery, personalized architecture, tissue engineering	Muliaditan et al.,^73^ Liu et al.,^74^ Peng et al.^75^	proof-of-concept	customized design; complex structure fabrication; promotes tissue regeneration

#### Prevent bleeding

The primary objective is to avoid any procedures that may cause mechanical damage. Perform all actions with the utmost gentleness, avoiding wiping. Select non-adherent, non-damaging moist wound dressings, such as hydrocolloid dressings or more advanced soft silicone contact layer dressings. These dressings seamlessly conform to the wound surface, effectively maintaining a moist wound bed while protecting newly formed granulation tissue. During dressing changes, they minimize disruption to the wound bed, reducing mechanical damage to fragile blood vessels and nerve endings, thereby lowering the risk of bleeding at its source. When faced with minor active bleeding, the selected dressing must also possess hemostatic properties. Alginate dressings demonstrate unique advantages in this scenario. They efficiently absorb exudate while releasing calcium (Ca) ions to activate the coagulation cascade, providing natural hemostatic properties. For minor bleeding, apply the alginate dressing directly over the site and apply gentle yet firm local pressure for 10 to 15 min to leverage its dual chemical and physical mechanisms for effective hemostasis. If bleeding persists despite these measures, timely escalation to medical intervention is required. This tiered approach, combining prevention and management, significantly reduces the frequency and severity of bleeding episodes, thereby alleviating patient distress and psychological anxiety.

#### Preventing pain

Systematic preemptive medication is the key foundation. Systematically administer analgesics 30–60 min prior to dressing changes to elevate pain thresholds and establish a “pharmacological barrier.”^7^ Pain-free precision techniques form the core practice. Thoroughly saturate old dressings with warm sterile saline until adhesions fully release, then gently remove them parallel to the wound surface. Prioritize irrigation and autolytic debridement to minimize mechanical irritation. Localized targeted analgesia delivers precise solutions. Post-dressing application of lidocaine-infused hydrogels or dressings blocks nerve signal transmission at the peripheral level, providing sustained, highly effective pain relief with minimal side effects. Humanized communication and environmental support serve as vital adjuncts. Thorough pre-procedure explanations, distraction techniques during procedures, and creating a warm, private environment alleviate patient anxiety and fear psychologically. Pain management for cancer-related wounds, particularly procedural pain from dressing changes, requires a systematic, multimodal, and prevention-focused proactive strategy. Advancing pain management to an earlier stage—blocking or reducing pain at its source through synergistic mechanisms—is the cornerstone of effective palliative care.

In summary, silver-containing dressings have become the cornerstone for managing infection and odor, and can be synergistically combined with activated charcoal dressings. For controlling significant exudate, highly absorbent dressings such as alginate and hydrophilic fiber dressings are the preferred choice. For bleeding and pain, soft silicone dressings enable pain-free dressing changes, while alginate dressings inherently possess hemostatic properties. Ultimately, successful dressing management is never a single intervention. It must be embedded within a comprehensive care framework encompassing debridement, antineoplastic therapy, and multidisciplinary collaboration. Through dynamic, individualized strategies, the ultimate goal of enhancing patient quality of life is achieved.

## Cutting-edge technologies and innovative strategies for cancer wound dressings

Currently, cutting-edge technologies and innovative strategies for cancer wound dressings are shifting from passive coverage to active management, centered on building intelligent therapeutic platforms that dynamically respond to the wound microenvironment.^76^ Technological innovations include: smart dressings that release antimicrobial agents or growth factors based on exudate pH levels; bioactive dressings integrating components such as chitosan and honey, offering potent anti-inflammatory properties alongside autolytic debridement capabilities; advanced foam and alginate dressings utilize super-absorbent mechanisms to lock in moisture, providing sustained dryness for wounds with extremely high exudate levels.^67^^,^^76^ Strategic innovations emphasize combination therapies, such as integrating NPWT with targeted dressings to synergistically manage exudate, control infection, and promote granulation tissue growth. These advancements collectively propel cancer wound care toward a new era of precision and individualization, aiming for comprehensive symptom control and enhanced patient quality of life.^68^

### Injectable hydrogels: From fillers to smart therapeutic platforms

Injectable hydrogels represent a breakthrough technology in cancer wound management, particularly suited for irregular cavitary wounds. These materials can be directly injected into the wound bed via syringe, forming a three-dimensional network structure *in situ* that conforms to the wound’s shape.^70^^,^^71^^,^^72^

**Figure 4:**
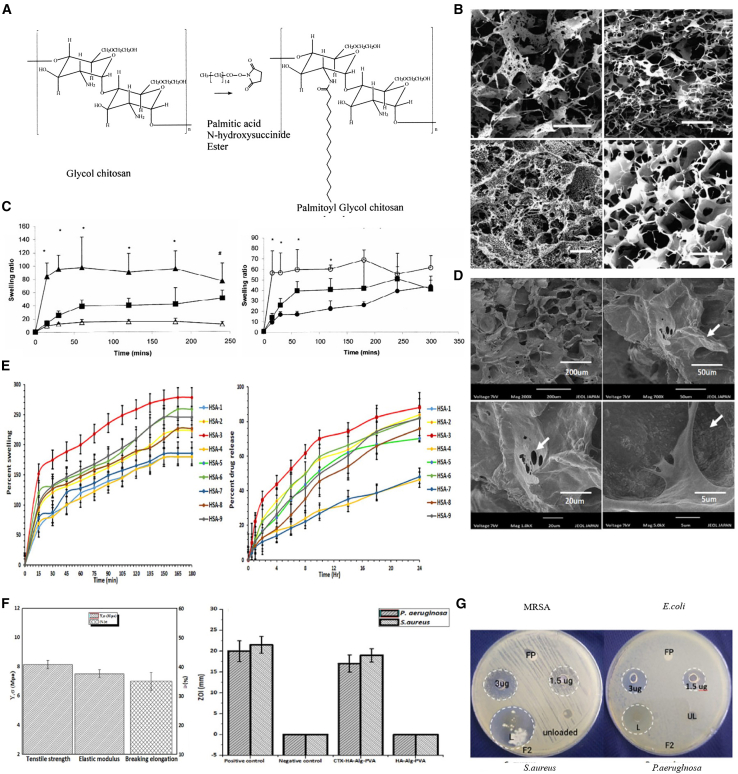
Hyaluronic acid-based hydrogels (A) Schematic diagram depicts the synthesis route for palmitoyl glycol chitosan. Image was reproduced from Moeini, A. et al.^76^ with permission. (B) Morphological characterization of palmitoyl glycol chitosan (GCP)-based hydrogels via scanning electron microscopy (scale bars, 200 μm). Image was reproduced from Moeini, A. et al.^76^ with permission. (C) Swelling profiles of GCP hydrogels showed comparable swelling. Image was reproduced from Moeini, A. et al.^76^ with permission. (D) Representative SEM images of HA-Alg-PVA hydrogel membranes captured at increasing magnifications, demonstrating the porous structure. Image was reproduced from Khaliq, T.et al.^56^ with permission. (E) Time-dependent swelling kinetics of HA-Alg-PVA hydrogel membranes in simulated physiological medium. Image was reproduced from Khaliq, T. et al.^56^ with permission. (F) Evaluation of the mechanical strength of HA-Alg-PVA hydrogel membranes. Image was reproduced from Khaliq, T.et al.^56^ with permission. (G) Inhibition zone diameters (mm) against *S. aureus* and *P. aeruginosa*. Image was reproduced from Khaliq, T.et al.^56^ with permission.

HA-based hydrogels, as natural components of the ECM, exhibit excellent biocompatibility and degradability.^57^ Functionalized HA can form stable structures through physical or chemical crosslinking and serve as a carrier for various therapeutic agents (e.g., chemotherapeutic drugs, immunomodulators, and growth factors).^8^ Studies demonstrate that HA hydrogels loaded with epirubicin maintain effective drug concentrations at tumor sites for over 72 h, significantly inhibiting local tumor progression while promoting wound epithelialization (Figure 4).^64^

Self-healing hydrogels possess internal dynamic covalent bonds (such as borate bonds and disulfide bonds) that enable them to restore structural integrity after mechanical disruption, a property particularly crucial in wound environments requiring frequent dressing changes.^8^ These hydrogels can integrate pH- or enzyme-responsive release mechanisms to deliver drugs precisely in response to specific signals within the tumor microenvironment, such as those triggered by MMPs and cathepsins.^64^^,^^67^

Nano-composite hydrogels incorporate functional nanomaterials—such as mesoporous silica nanoparticles and metal-organic frameworks—into polymer networks, significantly enhancing drug-loading capacity and mechanical properties (Table 3).^68^ Research demonstrates that PLGA nanoparticles loaded with paclitaxel, when embedded within thermosensitive hydrogels, enable sustained and localized chemotherapy delivery to cancerous wounds, markedly reducing systemic toxicity.^49^^,^^69^

**Table 3:** The types of nanoparticles

Nanoparticle type	Visual representation	Key features annotated	Therapeutic application in cancer wounds (as discussed in text)	Reference
Liposomes	spherical phospholipid bilayer with aqueous core	hydrophilic core (for drugs such as doxorubicin); hydrophobic bilayer (for drugs such as paclitaxel)	delivery of chemotherapeutics and immunomodulators; surface functionalization for targeting	Naseri et al.^68^
Polymeric Nanoparticles	solid spherical matrix	biodegradable polymer matrix (e.g., PLGA); encapsulated drug molecules	sustained release of anticancer drugs; incorporation into hydrogels for localized therapy	Moradian et al.^49^
Mesoporous Silica Nanoparticles (MSNs)	spherical particle with hexagonal pore channels	high surface area pores; drug-loaded pores; functionalized surface for “gatekeeping”	high-capacity drug loading; stimuli-responsive release (pH, enzyme); incorporated into nanocomposite hydrogels	Naseri et al.^68^
Metal Nanoparticles	solid spherical core (e.g., gold)	gold nanospheres/rods; surface plasmon resonance effect	photothermal therapy (NIR light → heat); synergistic photothermal-chemo combinations	Naseri et al.^68^
Carbon-Based Nanomaterials	sheet-like (graphene oxide) or spherical (carbon dots)	graphene oxide sheet with drug molecules; fluorescent carbon dot	drug delivery via π-π stacking; photothermal conversion; antioxidant activity (cerium-doped carbon dots)	Naseri et al.^68^

A critical advantage of injectable hydrogels, particularly for cancerous wounds with irregular geometries, is their ability to conform to and fill complex three-dimensional spaces. Unlike preformed dressings that may bridge across cavities or fail to contact undermined wound edges, injectable formulations flow into every recess, sinus tract, and crevice, establishing intimate and continuous contact with the entire wound bed. This space-filling property serves multiple therapeutic functions. First, it eliminates dead space where exudate could accumulate and bacteria could proliferate, thereby reducing infection risk. Second, it ensures uniform distribution of any incorporated therapeutic agents throughout the wound, maximizing treatment efficacy. Third, the hydrated three-dimensional network provides a physical scaffold that supports cell infiltration, angiogenesis, and granulation tissue formation from all wound surfaces, rather than only from the superficial wound bed. Histological evidence confirms that wounds treated with space-filling hydrogels exhibit more uniform re-epithelialization, organized collagen deposition, and enhanced neovascularization compared to those treated with conventional dressings that fail to fill the wound volume.

### Bioactive materials: A multifunctional platform beyond antimicrobial properties

Traditional biomaterials primarily serve as structural supports or passive fillers, whereas the new generation of silicate bioactive materials—such as bioactive glass and Ca-silicate ceramics—are demonstrating remarkable bioactivity that transcends their fundamental functions.^72^ They are emerging as multifunctional platforms capable of actively regulating the body’s microenvironment.^60^ Their core mechanism lies in the ability to induce the formation of a hydroxyapatite layer—a bone-like phosphate layer—on their surface when implanted *in vivo* and exposed to tissue fluid.^71^ This structure closely mimics the mineral composition of natural human bone, providing an ideal adhesion and proliferation interface for surrounding osteoblasts and other cells, thereby strongly promoting tissue regeneration. Moreover, it functions as a “signal relay station,” initiating deeper biological dialogues.^71^^,^^72^

**Figure 5:**
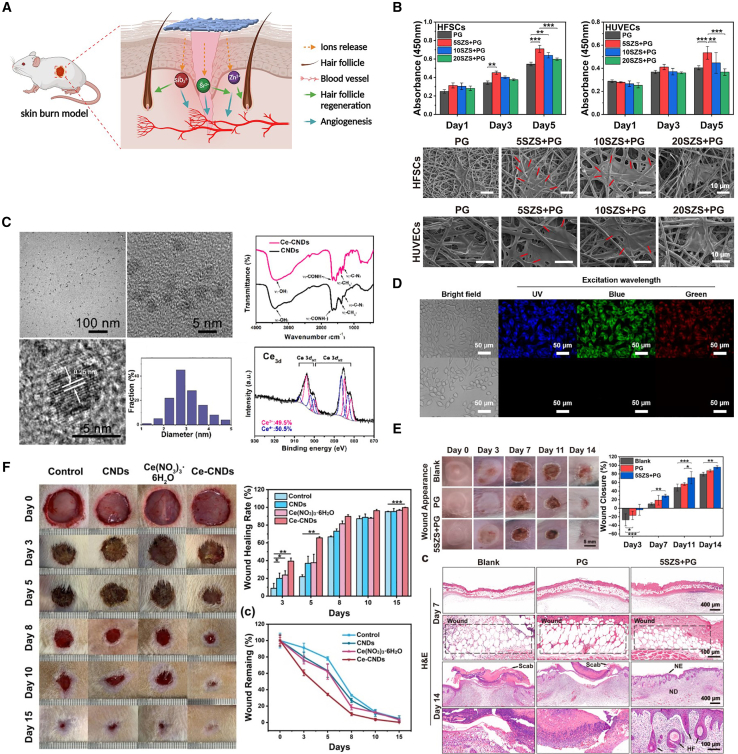
Bioactive materials (A) A Sr-Zn-Si bioceramic-incorporated PCL/gelatin electrospun membrane (5SZS + PG) showed promise for treating skin burn wounds and promoting hair follicle regeneration. Image was reproduced from Yu, J. et al.^77^ with permission. (B) *In vitro* biocompatibility was assessed by culturing rat hair follicle stem cells (HFSCs) and human umbilical vein endothelial cells (HUVECs) on various composite electrospun membranes, with results showing cell viability, morphological changes (pseudopodia indicated by red arrows), and statistical significance. Image was reproduced from Yu, J. et al.^77^ with permission. (C) The proliferation of HUVECs and fibroblasts was measured after 1, 3, and 7 days of exposure to ionic extracts from four types of bioactive glass microfibers. Image was reproduced from Zhang, M. et al.^78^ with permission. (D) A comprehensive characterization of cerium-doped carbon nanodots (Ce-CNDs) was presented with both excellent hydrophilicity and antioxidant activity. Image was reproduced from Zhang, M. et al.^78^ with permission. (E) Fluorescence microscopy images compare L929 cells labeled with Ce-CNDs, indicating the superior photostability of Ce-CNDs. Image was reproduced from Zhang, M. et al.^78^ with permission. (F) An *in vivo* burn wound model evaluated different treatments, displaying wound photographs over 14 days, closure rates, and H&E-stained histology showing that the 5SZS + PG group significantly facilitated the healing process of skin burn wounds. Image was reproduced from Yu, J. et al.^77^ with permission. (G) The wound healing progression for full-thickness excision wounds treated with Ce-CNDs was shown to validate the efficacy of Ce-CNDs *in vivo*. Image was reproduced from Zhang, M. et al.^78^ with permission.

More crucially, during their degradation, these materials continuously and controllably release bioactive ions such as silicon (Si), Ca, and strontium (Sr) (Figure 5).^73^^,^^77^ Far from being mere metabolic byproducts, these ions act as potent signaling molecules capable of precisely modulating immune system responses. One prominent function is regulating macrophage polarization. Macrophages are central to the immune system, yet their function becomes imbalanced in many pathological states. In environments such as tumors or chronic inflammation, pro-inflammatory M1 phenotype macrophages often become overactive, exacerbating tissue damage and hindering repair. Bioactive ions released from silicate materials can skillfully convert macrophages from the destructive M1 phenotype to the anti-inflammatory, repair-promoting M2 phenotype.^79^ This polarization shift effectively improves the tumor microenvironment, suppresses excessive inflammatory responses, and creates conditions more conducive to tissue regeneration and healing.^80^

Through meticulous material design, particularly by doping specific functional ions, these silicate platforms can be further “empowered” with additional, precisely targeted therapeutic functions, achieving synergistic effects through “multifunctional materials.” For instance, copper (Cu)-doped bioactive glass exemplifies this approach. Cu ions not only possess inherent antibacterial properties but also catalyze the Fenton reaction. Within acidic microenvironments such as tumors, this reaction converts hydrogen peroxide into highly toxic hydroxyl radicals, generating substantial reactive oxygen species.^78^ This chemotherapeutic approach efficiently eliminates bacteria while inflicting lethal oxidative damage on tumor cells, delivering dual antibacterial and antitumor effects. Conversely, cerium-doped (Ce) materials exhibit potent antioxidant potential. Cerium ions, with their unique variable valence states (Ce^3+^/Ce^4+^), mimic the activity of superoxide dismutase and catalase in the human body, efficiently scavenging excess ROS.^81^ This property is crucial for alleviating oxidative stress—a common condition in tumors or chronic wounds caused by ROS accumulation. It helps protect healthy cells, maintain microenvironmental stability, and thereby support the repair process.^82^

Beyond injectable hydrogels, preformed porous scaffolds represent another approach to space-filling wound management. Materials such as 3D-printed polymer scaffolds, freeze-dried collagen sponges, and electrospun nanofiber mats are engineered with defined pore architectures that physically occupy the wound volume while providing a template for tissue ingrowth. The interconnected porosity of these scaffolds (typically 50–500 μm pore size) allows for cell migration, nutrient diffusion, and vascular infiltration throughout the entire scaffold volume, effectively transforming the dressing from a temporary covering into a regenerative template. When combined with bioactive ions (e.g., Sr^2+^, Zn^2+^, and Cu^2+^) or growth factors, these space-filling scaffolds actively direct tissue regeneration while simultaneously delivering localized therapy. Micro-CT imaging has demonstrated that such scaffolds maintain their structural integrity and pore architecture within the wound over time, supporting gradual tissue replacement as the scaffold degrades.

Silicate bioactive materials have evolved from mere “bone repairers” into multifunctional platforms integrating tissue regeneration, immune modulation, and precision therapy. This advancement paves the way for broad applications of biomaterials in complex fields such as regenerative medicine and tumor treatment.

### Local combined treatment strategy: Synergistic enhancement model

**Figure 6:**
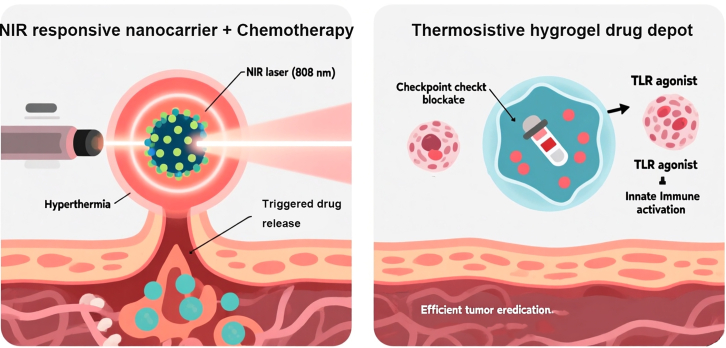
Schematic illustration of localized combined treatment strategies: synergistic enhancement through photothermal-chemotherapy and immune modulation

When confronting complex pathological environments such as cancerous wounds, monotherapy often proves inadequate. Consequently, synergistic strategies that organically integrate two or more treatment modalities at the local lesion site have emerged as a frontier in current research. This approach addresses the limitations of individual therapeutic modalities by leveraging their synergistic interaction, resulting in a combined effect that is substantially greater than the sum of their independent actions. This synergistic model manifests primarily in the following aspects (Figure 6).

#### Photothermal-chemotherapy combination therapy

**Figure 7:**
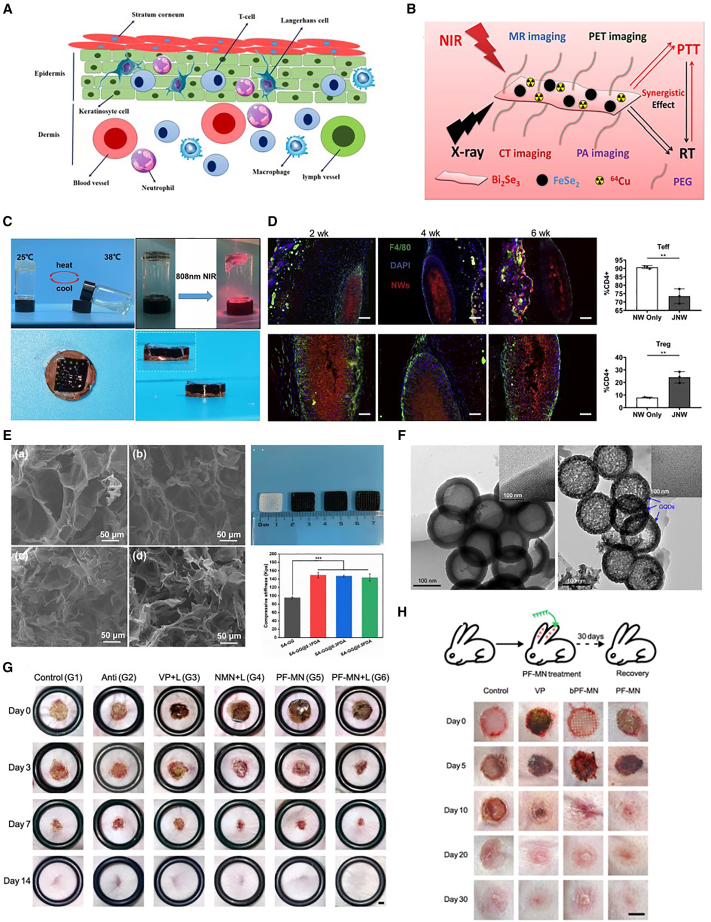
Combination therapy (A) This schematic drawing illustrates the types and layered distribution of skin cells involved in immunomodulation. Image was reproduced from Heydari, P. et al.^84^ with permission. (B) A schematic depicts the core-shell FeSe_2_/Bi_2_Se_3_ nanocomposite designed for multimodal imaging and combined photothermal-radiation therapy of tumors. Adapted from Wang, J.^82^ (C) Photographs show the temperature- and NIR-induced phase transition of a thermosensitive hydrogel, as well as front and side views of a sodium alginate (SA)-gellan gum (GG)@PDA + doxorubicin (DOX) heterogeneous scaffold. Image was reproduced from Xu, L. et al.^85^ with permission. (D) *In vivo* clearance of polycaprolactone (PCL) nanowires and *in vitro* activation of JNWs by lymph-node-derived lymphocytes cultured with IL-2 (scale bars, 100 μm and 200 μm). Image was reproduced from Heydari, P. et al.^84^ with permission. (E) SEM images and macroscopic photographs display 3D-printed SA-GG hydrogel scaffolds containing different concentrations of polydopamine. Image was reproduced from Xu, L. et al.^85^ with permission. (F) TEM images characterize hollow mesoporous carbon nanoparticles (HMCN) and the resulting HA-HMCN(DOX)@GQDs composite, with insets highlighting the mesoporous shell and graphene quantum dots (GQDs). Image was reproduced from Wang, J.et al.^82^ with permission. (G) A core-shell structured microneedle patch with programmed functions promotes healing of MRSA-infected diabetic wounds (scale bars, 2 mm). Image was reproduced from Zhang, Y.et al.^86^ with permission. (H) Experimental schematic of PF-MN-assisted scarless wound healing in a rabbit ear model. (b) Representative photographs of wounds receiving different treatments at indicated time points. Scale bars, 5 mm. Image was reproduced from Zhang, Y.et al.^86^ with permission.

This strategy ingeniously integrates photothermal therapy with chemotherapy.^83^ Its core lies in utilizing near-infrared light-responsive materials—such as two-dimensional black phosphorus quantum dots and MXene—as carriers and effectors. When exposed to near-infrared laser light at specific wavelengths, these materials efficiently convert light energy into heat, causing rapid local temperature elevation at the tumor site (Figure 7).^85^ This localized hyperthermia directly causes protein denaturation and cell membrane damage in tumor cells, achieving physical ablation. However, its more sophisticated effect lies in enhancing chemotherapy efficacy^74^: On one hand, heat increases vascular permeability and cell membrane fluidity in tumor tissues, significantly promoting the penetration and uptake of chemotherapeutic drugs into cells; on the other hand, thermal energy suppresses the activity of drug efflux pumps in tumor cells, effectively reversing drug resistance. Studies demonstrate that loading classic chemotherapeutic agents such as doxorubicin onto highly efficient melanin nanoparticles for photothermal conversion enables not only controlled, rapid drug release under laser irradiation but also enhances its cytotoxicity through thermotherapy. This produces a synergistic killing effect on inherently drug-resistant tumor cells, effectively preventing tumor recurrence.^87^

### Immune modulation strategies

The onset and progression of cancer are closely linked to immunosuppression within the tumor microenvironment, making local immune modulation a revolutionary therapeutic approach.^86^^,^^88^ This strategy aims to transform the “immune-cold environment” surrounding tumors into an “immune-hot environment” with anti-tumor activity. To achieve this goal, researchers have engineered novel biomaterials—such as hydrogels—capable of locally delivering immunomodulators.^72^ For instance, thermosensitive hydrogels co-load immune checkpoint inhibitors (e.g., anti-PD-L1 antibodies) that “release” the brakes on immune cells (particularly T cells) with Toll-like receptor agonists that “press the accelerator” to activate innate immunity. When injected or implanted into cancerous wounds, these hydrogels form a sustained-release “drug depot” at the tumor site.^36^^,^^84^ This localized delivery offers significant advantages: It achieves therapeutic concentrations directly at the lesion, powerfully activates T cells to enhance their infiltration and killing capabilities, while minimizing systemic administration-related severe immune-related adverse events (such as pneumonia or colitis). Animal studies have confirmed that this localized immunotherapy regimen significantly suppresses tumor growth and induces durable immune memory, effectively prolonging overall survival in model animals.^89^

The localized combined therapy strategy exerts synergistic effects through multiple mechanisms in both temporal and spatial dimensions. It not only directly eliminates tumor cells but also fundamentally alters the unfavorable local microenvironment, offering a promising solution for achieving efficient, low-toxicity treatment of complex conditions such as cancerous wounds.

## Clinical translation challenges and future development directions

### Barriers from laboratory to clinical practice

Despite significant advances in novel wound dressings based on advanced materials demonstrated in laboratory studies, showcasing immense therapeutic potential, their clinical translation from the laboratory bench to the patient’s bedside still faces multiple formidable challenges. These obstacles span multiple dimensions, including safety, manufacturing, and health economics, presenting issues that developers must confront and resolve.

First and foremost, a comprehensive safety assessment is an essential prerequisite, one that far exceeds the complexity of traditional dressings. For innovative dressings incorporating engineered nanomaterials, particular attention must be paid to the long-term effects of their biocompatibility. This includes: whether nanomaterials have the potential for long-term retention after wound healing, whether they migrate to distant organs and produce cumulative effects; whether the chemical properties, metabolic pathways, and potential organ toxicity of their degradation products are well-defined; and whether these materials and their degradation products could unexpectedly activate or suppress the immune system, triggering uncontrolled inflammatory responses or immunogenicity issues. All these aspects require validation through more sophisticated and extended *in vitro* and *in vivo* experiments.

Second, standardizing and scaling production processes remains a bottleneck for industrialization. Many laboratory synthesis methods—such as preparing nanomaterials or constructing complex drug delivery systems—are often limited to microgram or milligram scales and exhibit poor reproducibility. When scaling up to kilogram-level production for clinical trials or market supply, ensuring high consistency across batches in critical parameters—including composition, purity, particle size, porosity, and drug release kinetics—presents a monumental engineering challenge. For composite “combination products” comprising biomaterials, active cells, and drugs, establishing rigorous, multidimensional quality control standards and testing protocols demands exceptionally stringent production conditions and quality assurance systems.

Finally, against the backdrop of limited healthcare resources, cost-effectiveness analysis is particularly crucial. The research, development, and production costs of innovative dressings typically far exceed those of traditional dressings. Therefore, their high cost must be balanced by clear clinical benefits. Decision-makers and payers (such as health insurance agencies) require compelling evidence demonstrating that these dressings can reduce overall healthcare expenditures by accelerating healing rates, significantly decreasing dressing change frequency, effectively lowering the incidence of serious complications such as infections or amputations, and improving patient quality of life. To this end, actively conducting real-world studies to gather efficacy and cost-effectiveness data from clinical practice, coupled with rigorous health economic research, is crucial for demonstrating their value, gaining payer acceptance, and ultimately achieving widespread clinical adoption.

### Future research priorities and development trends

The complexity and distinctiveness of cancerous wounds necessitate continuously evolving management strategies. Future research will transcend simple coverage and absorption, advancing toward smarter, more precise, and more regenerative approaches. Specifically, key developmental trends will focus on the following cutting-edge directions.

(1) Personalized Precision Medicine: Shifting from “empirical treatment” to “data-driven” intervention

Future cancer wound management will increasingly shift toward personalization. The core strategy lies in conducting in-depth multi-omics analysis of wound exudate, treating it as an information-rich “liquid biopsy” sample.^90^ By analyzing specific biomarkers within it—such as cytokine profiles reflecting immune status, microbiomes revealing microbial balance, and tumor-derived exosomes carrying key signals—we can decode the wound’s real-time molecular characteristics.^91^^,^^92^ Based on this, clinicians can select or customize dressings with precision-guided accuracy: For wounds highly expressing specific inflammatory factors, dressings loaded with corresponding neutralizing antibodies can be applied; for microbial infections carrying specific resistance genes, dressings releasing targeted antibiotics can be deployed. This approach aims to achieve precision interventions based on molecular profiling, maximizing therapeutic efficacy while minimizing side effects.^9^^,^^10^^,^^70^

(2) Closed-Loop Intelligent System: Building a Dynamic Adaptive Wound Management Platform

Closed-loop intelligent systems integrating microelectromechanical systems with advanced biomaterials represent the future of smart wound management.^93^ These systems continuously and non-invasively monitor local wound parameters—such as pH (acidic environments indicating infection or tumor progression), temperature (reflecting inflammation levels), specific bacterial loads, and key biomarkers—by embedding micro-sensors within dressings.^94^^,^^95^ Collected data are processed through a feedback control system, automatically triggering pre-programmed therapeutic responses.^96^ For instance, when sensors detect elevated levels of enzymes associated with bacterial biofilms, the system can release corresponding enzyme inhibitors or antibiotics. If pH drops significantly, buffering agents or chemotherapeutic drugs are deployed. This “monitor-analyze-intervene” closed-loop approach enables truly dynamic, automated wound management, drastically reducing delays and uncertainties associated with human intervention.^75^^,^^97^

(3) Tissue Engineering and Regenerative Strategies: From Passive Control to Active Reconstruction

In the long term, ideal dressings must not only control tumors and infections but also guide the regeneration of functional tissues.^56^^,^^98^ This will depend on the deep integration of tissue engineering and regenerative medicine technologies.^99^ Future advanced dressings may serve as scaffolds loaded with genetically engineered mesenchymal stem cells or induced pluripotent stem cells. These cells can not only secrete abundant growth factors to promote angiogenesis and cell migration but also be engineered via gene editing technologies (such as CRISPR-Cas9) to overexpress specific anti-tumor proteins or immunomodulatory factors. This approach simultaneously promotes granulation tissue growth while precisely suppressing local tumor cell activity. This strategy aims to overcome the dilemma of “repair without tumor control” or “tumor control without repair,” achieving synergistic advancement in tumor control and functional tissue reconstruction.^16^^,^^100^

(4) Standardization of Clinical Endpoints: Establishing a Comprehensive, Patient-Centered Efficacy Evaluation Framework

To objectively evaluate the value of these innovative technologies, standardizing clinical endpoints in cancer wound research is crucial.^101^^,^^102^ Currently, relying solely on single metrics such as “wound area reduction rate” is insufficient to reflect comprehensive efficacy. Future industry consensus is needed to establish a multidimensional assessment framework.^103^ This should include: Objective clinical indicators such as complete healing rate, infection control time, and tumor recurrence rate. Patient-reported outcomes such as pain intensity using visual analog scales, odor, and the impact of exudate on daily life—factors directly related to patients’ dignity in living. Health economics metrics such as quality-adjusted life years (QALYs), which comprehensively evaluate a treatment’s value in extending life and improving quality, provide critical guidance for healthcare resource allocation.^104^ Establishing such a comprehensive evaluation framework will provide a fair benchmark for comparing different therapies and accelerate the clinical adoption of genuinely effective innovative dressings.

### Surgical interventions and integration with advanced biomaterials

Surgical management of cancerous wounds encompasses several scenarios: palliative debulking to reduce tumor burden and symptom severity, resection of locally advanced cancers with curative intent, and reconstructive procedures to close surgical defects. Each of these scenarios presents unique challenges and opportunities for integration with advanced biomaterial-based wound management technologies.

Palliative Debulking Surgery: In patients with advanced disease, surgical reduction of the tumor mass can alleviate pressure on surrounding tissues, reduce exudate and odor, and improve the efficacy of subsequent topical therapies. However, the resulting surgical wound remains at high risk for poor healing, infection, and recurrence due to the underlying malignant and compromised tissue environment. Modern biomaterial dressings play a crucial role in the postoperative management of these wounds.

Curative-Intent Resection and Reconstruction: For locally advanced cancers where complete surgical resection is attempted, the creation of large tissue defects often necessitates reconstructive surgery, including skin grafts, local flaps, or free vascularized tissue transfer. Postoperative wound complications are exceptionally high in this population, with reported rates of 44% for primary closure, 57% for skin grafting, and 80% for flap reconstruction. Factors such as neoadjuvant radiotherapy, patient comorbidities (BMI >25, smoking), and high-grade lesions further compound the risk. Here, advanced biomaterials are being integrated directly into the surgical strategy. For example:

Biosynthetic meshes for perineal reconstruction after abdominoperineal resection have been shown to reduce wound dehiscence (1 vs. 9 cases, *p* = 0.033), accelerate healing (16 vs. 24 days, *p* = 0.015), and enable earlier initiation of adjuvant therapy (26 vs. 70 days, *p* = 0.003) compared to conventional procedures.

NPWT applied to closed surgical incisions (incisional NPWT) has demonstrated potential in reducing superficial surgical site infections after high-risk procedures such as pancreaticoduodenectomy for pancreatic cancer. However, its application directly over malignant wounds requires careful assessment due to bleeding risks.

Tissue-engineered scaffolds and cell-laden hydrogels represent the frontier of surgical reconstruction, aiming to regenerate functional tissue while simultaneously delivering localized anticancer or immunomodulatory therapy.

Management of Intraoperative and Postoperative Bleeding: Surgical manipulation of cancerous wounds carries a significant risk of bleeding due to the fragile, abnormal tumor vasculature. Modern hemostatic strategies align surgical and biomaterial approaches. Intraoperatively, surgeons may employ cauterization or ligation of bleeding vessels. Postoperatively, hemostatic dressings such as alginates (which release Ca ions to activate coagulation), oxidized regenerated cellulose, and topical agents such as adrenaline (under strict supervision) provide localized control. Non-adherent silicone dressings are essential to prevent re-bleeding during dressing changes by minimizing trauma to the surgical site.

Preoperative Optimization: Emerging evidence suggests that preoperative interventions can improve surgical outcomes. A recent clinical trial is investigating whether preoperative bacterial decolonization using topical chlorhexidine and mupirocin reduces infection rates in skin cancer surgery wounds left to heal by secondary intention.

## Conclusions and outlook

Cancer wound management is undergoing a paradigm shift from palliative symptom control to disease-modifying treatment. Modern dressing technologies, particularly injectable hydrogels, nanocomposites, and bioactive materials, have demonstrated immense potential beyond traditional dressings to simultaneously address the dual challenges of symptom management and disease regulation.^105^

Future successful cancer wound management strategies will inevitably become more personalized, multifunctional, and integrated, combining the latest advances in precision medicine, materials science, and tumor immunology.^106^ These innovative technologies hold promise for significantly improving the quality of life for patients with advanced cancer and offering novel solutions to this highly challenging clinical problem.^107^

With the conduct of more high-quality clinical studies and the continued maturation of these technologies, cancer wound dressings are poised to transition from supportive care tools to active therapeutic platforms in the near future, ultimately providing patients with more effective and comprehensive care.^13^^,^^104^

The clinical management of cancer wounds is undergoing a profound paradigm shift: moving from past palliative symptom control focused on cleansing and covering toward disease-modifying therapies aimed at fundamentally altering wound biology. This transformation is driven by the convergence of materials science, nanotechnology, and tumor biology.^108^ Modern advanced dressing technologies, particularly injectable hydrogels, nanocomposites, and bioactive materials, have demonstrated immense potential beyond traditional dressings. These are no longer passive coverings but active therapeutic platforms capable of simultaneously addressing dual challenges: symptom management (e.g., controlling exudate, bleeding, and odor) and disease modulation (e.g., inhibiting tumor proliferation, regulating the immune microenvironment, combating infection). They offer unprecedented solutions to this complex clinical challenge.^19^^,^^109^

Looking ahead, successful cancer wound management strategies will inevitably advance in depth toward personalization, multifunctionality, and integration. First, personalized precision medicine will achieve tailored interventions by analyzing the molecular profiles of wound exudate, transforming dressings from “generic” products into “customized” solutions.^68^ Second, multifunctionality will manifest through integrating multiple therapeutic modalities onto a single platform. This includes synergistically combining photothermal therapy, chemoimmunotherapy, and bioactive ion therapy to deliver a “combination punch” targeting the multifactorial etiology of cancer wounds.^13^ Ultimately, these functions will be managed through a closed-loop system via integrated intelligent platforms. These systems will incorporate micro-sensors for real-time wound monitoring and automatically adjust drug release based on feedback, forming an adaptive dynamic therapeutic loop.^110^

Realizing this ambitious vision hinges on deep collaboration across multiple disciplines, including precision medicine, materials science, and tumor immunology. As more meticulously designed, mechanism-driven preclinical studies advance, accompanied by a series of high-quality clinical trials and the continuous maturation of technologies, we have every reason to believe that cancer wound dressings will soon achieve their ultimate transformation—evolving from an auxiliary nursing tool into a powerful, proactive disease management platform.^2^ This platform will not only control local disease progression but also modulate systemic immune states through localized interventions. It may even synergize with systemic therapies such as immune checkpoint inhibitors, thereby extending patient survival while significantly enhancing quality of life and dignity. Ultimately, these innovations promise to deliver more effective, humane, and comprehensive care for patients with advanced cancer, illuminating previously neglected corners of cancer wound treatment.

## Acknowledgments

This work was jointly supported by the General Humanities Research Project of the Liaoning Provincial Department of Education, no. JYTMS20230145.

## Declaration of interests

The authors declare no competing interests.

## Declaration of generative AI and AI-assisted technologies in the writing process

During the preparation of this work, the authors used DeepSeek to improve the readability and language of the manuscript. After using this tool/service, the authors reviewed and edited the content as needed and take full responsibility for the content of the published article.
